# Effects of Nanoscale precipitates on mechanical properties, corrosion resistance, and biocompatibility in Zn-Mn alloy

**DOI:** 10.1038/s41598-025-89748-w

**Published:** 2025-02-14

**Authors:** Cuilan Dong, Zikun Liao, Yanyi Yin, Yinzhi Yi, Guanghui Zhu, Tuquan Zheng, Qian Tan, Yonghong Xie

**Affiliations:** 1 Nursing Department, Hunan Provincial Key Laboratory of Pediatric Orthopedics, Hunan Children’s Hospital, Hunan, 410007 China; 2 Orthopedic Department, Hunan Provincial Key Laboratory of Pediatric Orthopedics, Hunan Children’s Hospital, Hunan, 410007 China; 3 Department of Spine, The Third Affiliated Hospital of Guangzhou Medical University, Guangzhou, 510150 China; 4 Department of Neurosurgery, Nanfang Hospital, Southern Medical University, Guangzhou, 510515 Guangdong China

**Keywords:** Zn-Mn alloys, MnZn_13_ phase, Mechanical properties, Corrosion mechanism, Biocompatibility, Biomedical materials, Biomaterials - cells, Implants

## Abstract

Controlling degradation rate is essential for the biomedical application of biodegradable Zn alloys. Alloying with soluble elements is an effective way to regulate formation of second phases, which differ in potential from the Zn matrix. The potential difference exhibits positive or negative effects on corrosion resistance. This study successfully forms MnZn_13_ phase with nano size by altering ECAP temperature. Subsequently, MnZn_13_ phase promotes grain refinement, improvement of elongation, and corrosion resistance. Higher elongation in Zn-Mn alloy with MnZn_13_ phase is attributed to the grain boundary sliding, deformation twins in MnZn_13_ phase. Meanwhile, grain boundary corrosion in Zn-Mn alloy with MnZn_13_ phase is incomplete. Corrosion mode of Zn-Mn alloys without MnZn_13_ phase is dominated by grain boundary corrosion, accompanied by pitting corrosion. The increased corrosion resistance from MnZn_13_ phase stems from its higher potential than Zn matrix. Zn-Mn alloys with and without MnZn_13_ phase show a comparable cytocompatibility and osteogenic properties. Our findings provide an effective way to regulating mechanical properties and corrosion resistance of Zn alloys via controlling precipitation.

## Introduction

Guided bone regeneration (GBR) technology employs a membrane that acts as a barrier to separate soft tissues from areas of bone defect. This membrane specifically blocks fibroblasts from infiltrating the bone defect area, thus permitting osteoblast proliferation, which supports bone regeneration. Consequently, GBR is commonly applied in orthopedic and dental treatments. It is regarded as one of the most effective strategies for repairing bone defects^1–3^. An ideal GBR membrane requires good ductility alongside sufficient strength to offer robust support and efficient protection in restoring bone tissue functionality^4,5^. Additionally, it should possess a suitable degradation rate and high biocompatibility^6^. Yet, pure titanium (Ti)-based GBR membranes increasingly fall short of patient needs due to their non-biodegradable nature, often necessitating secondary removal surgery^7,8^. Bioabsorbable alternatives, such as collagen, polylactic acid (PLA), and other biodegradable polymers, are extensively utilized as GBR barriers for soft tissue. However, they often encounter issues related to insufficient mechanical strength, quick degradation, and limited osteogenic induction potential^9–11^.

Zinc (Zn) and its alloys exhibit suitable degradation rates and osteogenic potential, making them promising candidates for clinical applications in guided bone regeneration (GBR) membranes^12,13^. However, as a trace element with only 2–3 g present in adults, Zn^2+^ poses potential cytotoxicity concerns. For instance, human aortic smooth muscle cells (HASMC) can tolerate Zn²⁺ concentrations up to 5.2 µg/mL, while human dermal fibroblasts (HDF) have a tolerance threshold of 3.5 µg/mL, and human coronary artery endothelial cells (HCAEC) can withstand up to 6.5 µg/mL^14–16^. When Zn²⁺ concentrations surpass 6.5 µg/mL, both cell adhesion and proliferation are notably reduced, with levels around 14.4 ± 1.5 µg/mL potentially compromising the viability of mouse embryonic osteoblasts (MC3T3-E1)^13^. Ito et al. found that Zn²⁺ release at a concentration of 3 µg/mL in culture media does not induce cytotoxicity^17^. Therefore, precise balance of degradation rate with biocompatibility remains challenging.

Alloying with non-toxic elements is an effective strategy to control Zn alloy corrosion rates. Adding manganese (Mn) to Zn significantly enhances mechanical properties and adjusts the corrosion rate of Zn-Mn alloys^18–20^. According to the binary Zn-Mn phase diagram, solid solubility of Mn in Zn increases as the temperature increases^21^. Thus, the formation of the MnZn_13_ phase can be controlled through process parameters. Previous studies have demonstrated that the effects of Mn contents on corrosion rates of Zn-Mn alloys exhibit a “N-type” trend, showing an initial rise, a subsequent decrease, and final increase^22^. Altering Mn content in Zn-Mn alloys leads to the formation of MnZn_13_ phases with varying size. Coarse MnZn_13_ phases accelerate corrosion by galvanic effects due to the potential difference between Zn matrix and second phases^23^. Nanoscale MnZn_13_ phases precipitate from the supersaturated matrix, which are distributed in grains or at grain boundaries. These fine precipitates promote room-temperature superplasticity by inhibiting grain growth^24^. The corrosion morphologies of Zn-Mn alloys with nanoscale MnZn_13_ phases indicate that the uniform corrosion occurs at macroscopic level. Uniform degradation of implants is important for controlling release of metal ions. Therefore, it is necessary to investigate the effects of nanoscale MnZn_13_ phases on corrosion modes of Zn-Mn alloy.

This paper investigates Zn-Mn alloys with and without MnZn_13_ particles, examining their microstructure, mechanical properties, in vitro degradation behavior, cytotoxicity, and osteogenesis. Findings indicate that mechanical properties, corrosion rates, and biocompatibility can be optimized by regulating MnZn_13_ phase formation.

## Experimental procedure

### Preparation and characterization of Zn-Mn alloys

The Zn-Mn alloy, with an actual composition of Zn-0.41Mn wt%, was fabricated from high-purity Zn (99.99 wt%) and Mn (99.95 wt%). The alloy was melted in a low-carbon steel crucible under a carbon dioxide (CO₂) and sulfur hexafluoride (SF₆) (99:1) gas mixture and cast into a copper mold with dimensions of 200 mm × 50 mm × 50 mm. Cubic samples measuring 45 mm × 20 mm × 20 mm were extracted from the ingot for equal channel angular pressing (ECAP), performed at both ambient temperature and 200 ℃, with 12 passes.

Microstructural analysis of the Zn-Mn alloys was conducted using scanning electron microscopy (SEM). Elemental distribution was examined with an energy dispersive spectrometer (EDS) system (GENESIS 60 S model) attached to the SEM. Prior to SEM examination, alloy surfaces were mechanically ground, polished, and etched with a 4 vol% nitric acid solution in alcohol. To further analyze microstructural changes, electron backscatter diffraction (EBSD) and transmission electron microscopy (TEM) were performed. EBSD samples were prepared by mechanical grinding followed by electrochemical polishing with a 10 vol% perchloric acid (HClO₄) solution at -30 ℃ and 15 V. TEM samples were prepared using twin-jet electron polishing with a solution of 4 vol% HClO₄ in alcohol.

### Tensile tests

Standard tensile dog-bone specimens (ASTM E8/E8M-22) were sectioned along the ECAP direction using electrical discharge machining (EDM) and mechanically ground before testing. Tensile tests were conducted on a Zwick/Roell Z100 universal material testing machine (Germany), with total length, gauge length, width, and thickness of 40 mm, 10 mm, 2.4 mm, and 2 mm, respectively. The strain rate was set as 1 × 10⁻³ s⁻¹.

### Electrochemical and immersion tests

Electrochemical tests were conducted on a CHI660C electrochemical workstation using a traditional three-electrode setup in an electrochemical cell. Specimens were initially ground with SiC papers (grits 400 to 2000) and polished with a diamond suspension. The exposed sample surface area in simulated body fluid (SBF) was 10 mm × 10 mm, and testing was performed at 37℃. Potentiodynamic polarization (PDP) curves were obtained by sweeping the potential from − 1.6 V to -0.5 V (vs. saturated calomel electrode (SCE)) at a scan rate of 0.1 mV/s, allowing for the determination of self-corrosion potential (E_corr_) and corrosion current density (i_corr_). Electrochemical impedance spectroscopy (EIS) was conducted in the frequency range of 10^5^ Hz to 10^− 2^ Hz. The impedance data were analyzed with the ZView2 software package and fitted for equivalent curves.

For immersion tests, mechanically ground and polished samples were placed in centrifuge tubes filled with SBF, maintaining a metal area-to-solution volume ratio of 20 mL/cm². After a 30-day immersion, specimens were removed, rinsed with deionized water, and corrosion products were removed using a 200 g/L chromic acid solution. The corrosion rate (C, µm/year) was calculated as C = Δm/ρAt, where Δm is weight loss (mg), ρ is density (7.14 g/cm³), A is the exposed area (2.2 cm²), and t is time ( years).

### Cytotoxicity assessment

Endothelial cells (ECs) and bone mesenchymal stem cells (BMSCs), obtained from the Cell Bank of the Chinese Academy of Sciences, were used to assess cytotoxicity. Cells were cultured in Dulbecco’s Modified Eagle Medium (DMEM, Gibco) supplemented with 10% fetal bovine serum (FBS, Gibco) and maintained in a humidified incubator at 37 °C with 5% CO₂. Cell viability was evaluated using the Cell Counting Kit-8 (CCK-8) via an indirect contact method. Disc samples for cytotoxicity analysis were prepared similarly to those used in immersion tests. According to ISO 10993-12: 2012, extracts from Zn-Mn alloys were prepared by immersing disc samples in DMEM for 72 h, with a surface area to medium volume ratio of 1.25 cm²/mL. These extracts were then diluted to a 50% concentration for testing.

Cells were seeded in 96-well plates at 2000 cells per well. After a 24-hour incubation for cell attachment, the medium was replaced with diluted sample extracts, and cells were incubated for an additional 3 days. Subsequently, 10 µL of CCK-8 solution was added to each well. After 2 h, absorbance was measured at 450 nm using a microplate reader (iMARK, Bio-Rad, USA).

### ALP activity measurement, ALP staining and alizarin Red S staining

BMSCs were seeded into 6-well plates at a density of 6 × 10⁴ cells per well (3 × 10⁴ cells/mL, 2 mL per well). After 24 h of incubation, when cells reached 80% confluence, the culture medium was replaced with extracts from various groups. Zn-Mn alloy extracts served as the experimental group, while pure Ti extracts served as the control. Extracts were replenished every 48 h during incubation, with three wells allocated per group. After 7 and 14 days of osteogenic differentiation, the extracts were removed, and wells were rinsed three times with PBS.

For the detection of alkaline phosphatase (ALP) activity, cells were lysed using a 1% Triton X-100 solution. Three groups were set up for the experiment: the blank group (using ddH₂O), the detection group (comprising the cell sample), and the standard group (using a 0.1 mg/mL phenol standard solution). After conducting the colorimetric reaction, optical density (OD) at 520 nm was recorded with an enzyme-linked analyzer. A protein standard curve was generated, and the protein concentration in the cell samples was measured through the bicinchoninic acid assay (BCA). ALP activity for each group was then calculated as follows:

$$\begin{aligned} {\text{ALP activity (IU/mg total protein) = }} & {\text{ (Test OD - Blank OD)/(Standard OD - Blank OD)}} \\ & \times 0.{\text{1}}\,{\text{mg}}/{\text{mL}} \div {\text{Protein concentration (g protein/mL)}} \\ \end{aligned}$$

ALP and alizarin red staining (ARS) were performed to assess osteogenic potential. BMSCs cultured under the same differentiation conditions for 7 and 14 days were stained with ALP and ARS and examined under an optical microscope (Olympus Co., Ltd., Tokyo, Japan).

## Results

### Microstructures of Zn-Mn alloys

**Fig. 1:**
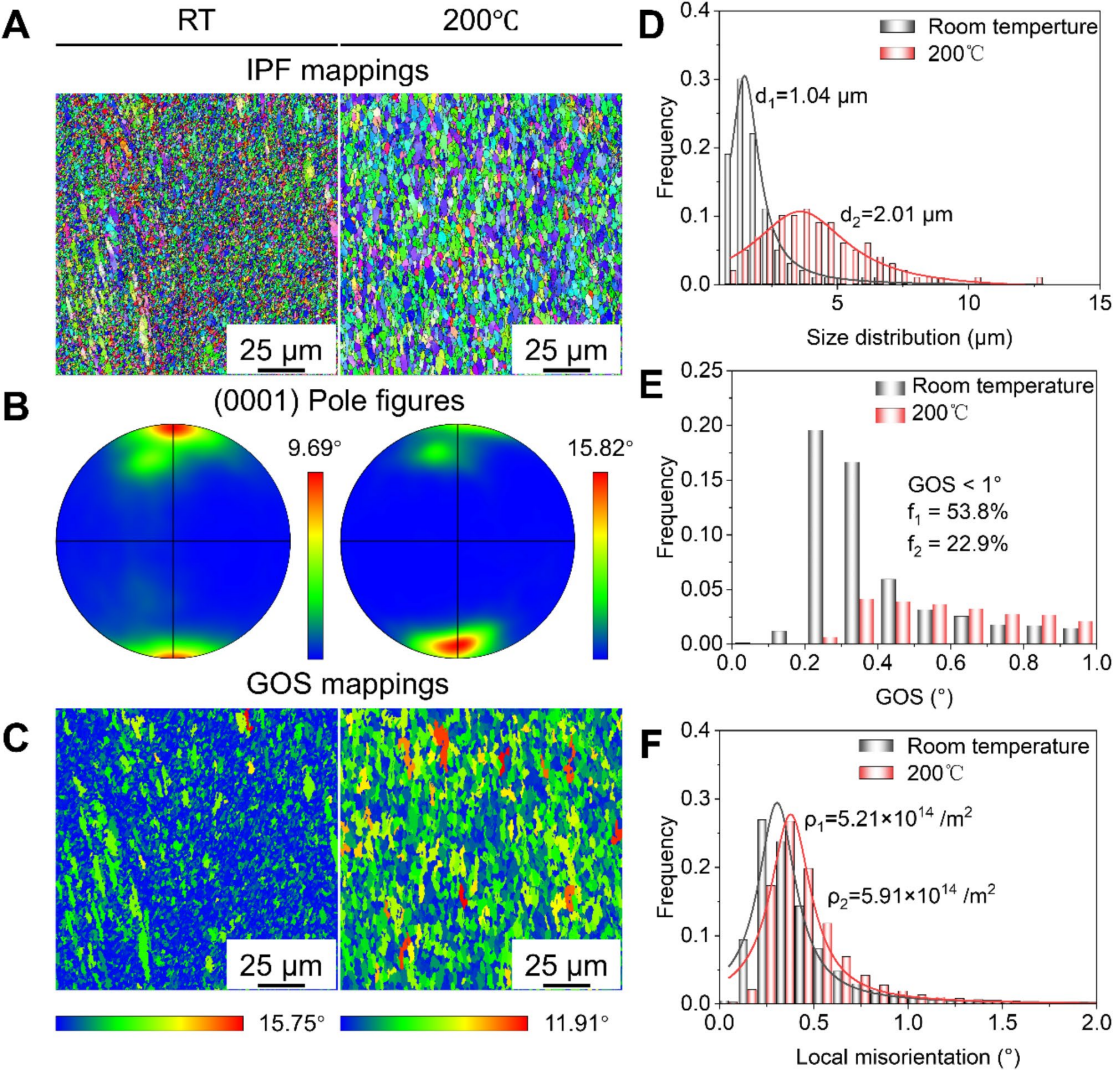
Microstructures of Zn-Mn alloys processed at different temperature. (**a**) IPF mappings. (**b**) Textures reflected by (0001) PF mappings. (**c**) GOS mappings. (**d**) Grain size distributions. (**e**) GOS distributions. (**f**) Dislocation densities.

Figure 1 presents the microstructures of Zn-Mn alloys processed at different temperatures. Inverse pole figures (IPFs) revealed a notable effect of processing temperature on the grain size of Zn-Mn alloys (Fig. 1a). Pole figure (PF) analysis indicated that processing temperature had minimal influences on texture orientation but showed that higher processing temperatures increased the texture intensity of the Zn-Mn alloy (Fig. 1b). Given Zn’s low melting point, the processing temperature exceeded its recrystallization temperature, allowing grain refinement through dynamic recrystallization (DRX). Typically, DRXed grains exhibited fewer dislocations, resulting in a low grain orientation spread (GOS) value (< 1°).

Figure 1d illustrates the grain size distribution of Zn-Mn alloy at various processing temperatures. The average grain size of Zn-Mn alloy (RT) was 1.04 μm, which increased to 2.01 μm of Zn-Mn alloy (200 °C). GOS distribution analysis showed that DRXed grain proportion in Zn-Mn alloy (RT) was 53.8%, decreasing to 22.9% at 200 °C. Additionally, the RT-processed Zn-Mn alloy contained a small number of elongated grains with high dislocation densities. Consequently, local misorientation results indicated that RT-processed Zn-Mn alloy exhibited a high dislocation density of 5.91 × 10^14^m^-2^.

**Fig. 2:**
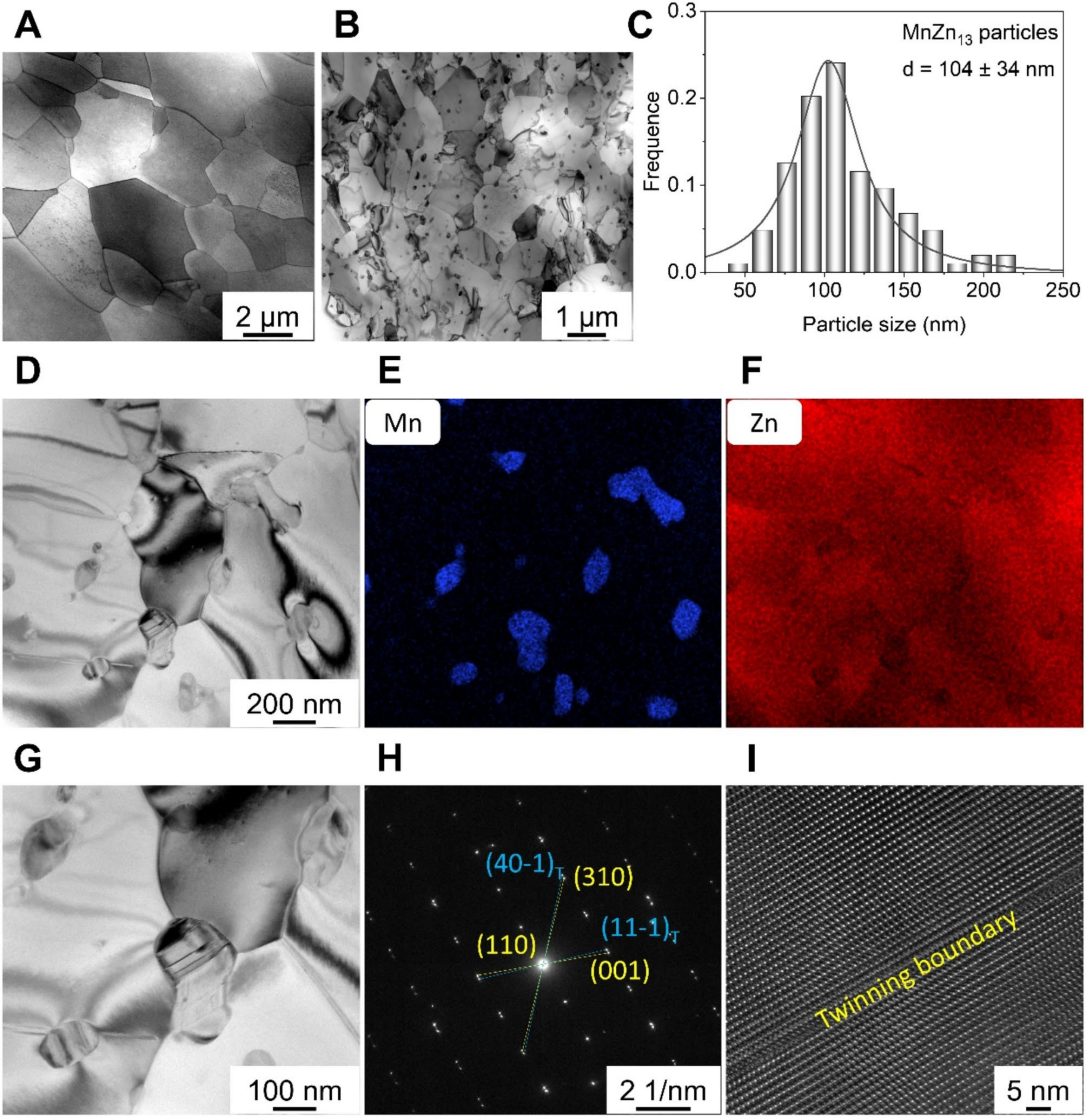
TEM analysis of Zn-Mn alloys. (**a**) 200℃, (**b**) RT. (**c**) Size distribution of MnZn_13_ phases. (**d**-**f**) MnZn_13_ phase and the corresponding EDS mappings. (**g**-**i**) Twins in MnZn_13_ phases, SAED image, and HR-TEM image.

After plastic deformation processing, the Zn-Mn alloy contained a significant number of equiaxed DRXed grains. Transmission electron microscopy (TEM) results showed that the Zn-Mn alloy processed at 200 °C consisted exclusively of DRXed grains (Fig. 2a). In contrast, the Zn-Mn alloy processed at room temperature exhibited numerous precipitates distributed within the grains and along grain boundaries (Fig. 2b). The average size of these precipitates was 104 ± 34 nm, substantially smaller than the DRXed grains (Fig. 2c). Elemental distribution analysis indicated that Mn was primarily located in the precipitates, consistent with the formation of the MnZn₁₃ phases, as shown in Fig. 2d, e, and f.

Additionally, twins were observed within the MnZn₁₃ phase, with thicknesses and interspaces less than 20 nm (Fig. 2g). Selective area electron diffraction (SAED) and high-resolution TEM patterns further confirmed that the twins in MnZn_13_ phases belong to {110} twins, as shown in Fig. 2h. Moreover, high-resolution TEM image (Fig. 2i) showed that the width of twin boundary was less than 5 nm. The occurrence of twins in the MnZn₁₃ phase was also documented in earlier literature, attributed to the good deformation ability of the MnZn₁₃ phase^25,26^.

### Mechanical behaviors of Zn-Mn alloys

**Fig. 3:**
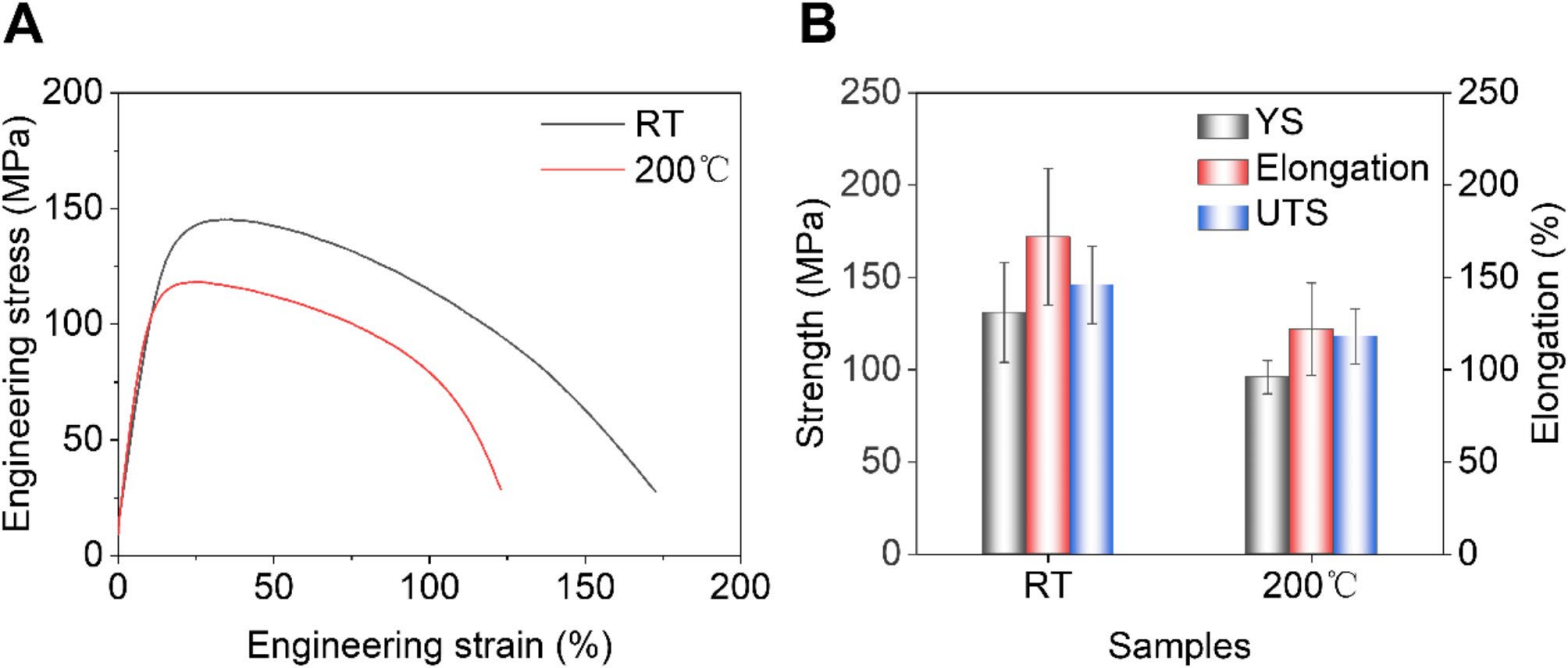
Mechanical behaviors of Zn-Mn alloys. (**a**) Typical tensile curves. (**b**) Comparison of strength and elongation.

The typical tensile curves of the Zn-Mn alloys are shown in Fig. 3a. As the processing temperature increased, the tensile strength and elongation of the Zn-Mn alloy decreased from 146 MPa and 172% to 118 MPa and 122%, respectively. The Zn-Mn alloy processed at RT exhibited higher tensile strength and elongation due to its smaller grain size and the high fraction of MnZn₁₃ phase at the grain boundaries, providing a synergistic strengthening effect from fine grains and precipitates. Figure 3b illustrates the differences in mechanical properties between Zn-Mn alloys processed at different temperatures, indicating that higher processing temperatures reduce the mechanical performance of Zn-Mn alloys.

### Corrosion behaviors of Zn-Mn alloys

**Fig. 4:**
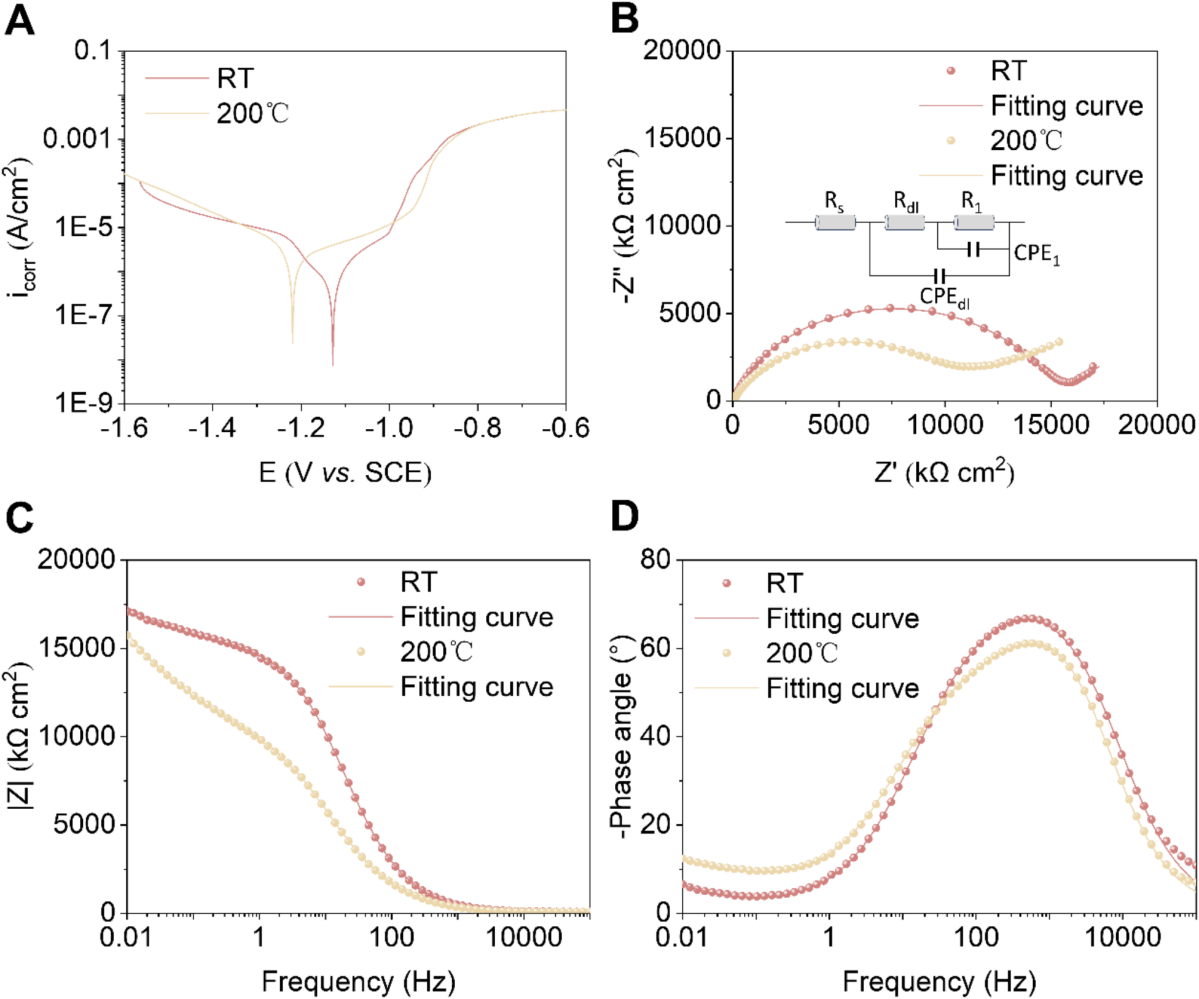
Electrochemical behaviors of Zn-Mn alloys. (**a**) PDP curves. (**b**) Nyquist plots and fitting circuit. (**c**) Bode diagram. (**d**) Phase angle vs. frequence.

Figure 4a presents the electrochemical polarization curves of Zn-Mn alloys. The corrosion potential (E_corr_) of Zn-Mn alloy processed at RT was − 1.1 ± 0.02 V, which was slightly higher than that of the Zn-Mn alloy processed at 200 °C (-1.2 ± 0.03 V), as shown in Table 1. However, the corrosion current density (i_corr_) was similar for both alloys, with values of 10.5 ± 0.8 µA/cm² for Zn-Mn alloy (RT) and 10.8 ± 1.6 µA/cm² for Zn-Mn alloy (200 °C). Figure 4b shows that the high-frequency loop radius in the impedance spectrum of the Zn-Mn alloy (RT) is larger than that of Zn-Mn alloy (200 °C), indicating better corrosion resistance.

In the simulation circuit, the charge transfer resistance (Rct) and corrosion product layer resistance (Rc) of the Zn-Mn alloy (RT) were higher than those of the alloy processed at 200 °C (Table 2), supporting its superior corrosion resistance. Figure 4c further shows that the |Z| value of the RT alloy decreases more gradually in the low-frequency region compared to the 200 °C alloy. Additionally, the maximum phase angle of the Zn-Mn alloy (RT) exceeded 60° (Fig. 4d), significantly higher than that of the 200 °C alloy, indicating a denser corrosion layer that effectively slowed the corrosion process^27^. These findings suggested that the Zn-Mn alloy (RT) exhibits superior corrosion resistance compared to the alloy processed at 200 °C.

**Table 1:** Corrosion data are obtained from PDP curves and immersion tests.

Alloys	E_corr_ (V vs. SCE)	i_corr_ (µA/cm^2^)	Corrosion rate (µm/year)
Zn-Mn (RT)	− 1.1 ± 0.02	10.5 ± 0.8	22.2 ± 6.7
Zn-Mn (200 °C)	− 1.2 ± 0.03	10.8 ± 1.6	29.4 ± 7.2

**Table 2:** Parameters in fitting circuit.

Samples	*R*_s_ (Ω cm^2^)	Rdl (Ω cm^2^)	CPE1 (10^− 6^ Ω-1 cm^− 2^ s^n1^)	R1 (Ω cm^2^)	CPE2 (10^− 6^ Ω-1 cm^− 2^ s^n2^)
Zn-Mn (RT)	69	7482	1.5	2034	5.6
Zn-Mn (200 °C)	74	6245	1.3	1915	7.4

Table 1 presents the corrosion rates of the alloys immersed in SBF solution for 30 days. The corrosion rates for the Zn-Mn alloy processed at room temperature (RT) and the Zn-Mn alloy processed at 200 °C were 22.2 ± 6.7 μm/y and 29.4 ± 7.2 μm/y, respectively. The corrosion rate of the Zn-Mn alloy (RT) was 21% lower than that of the Zn-Mn alloy (200 °C), consistent with the electrochemical results.

**Fig. 5:**
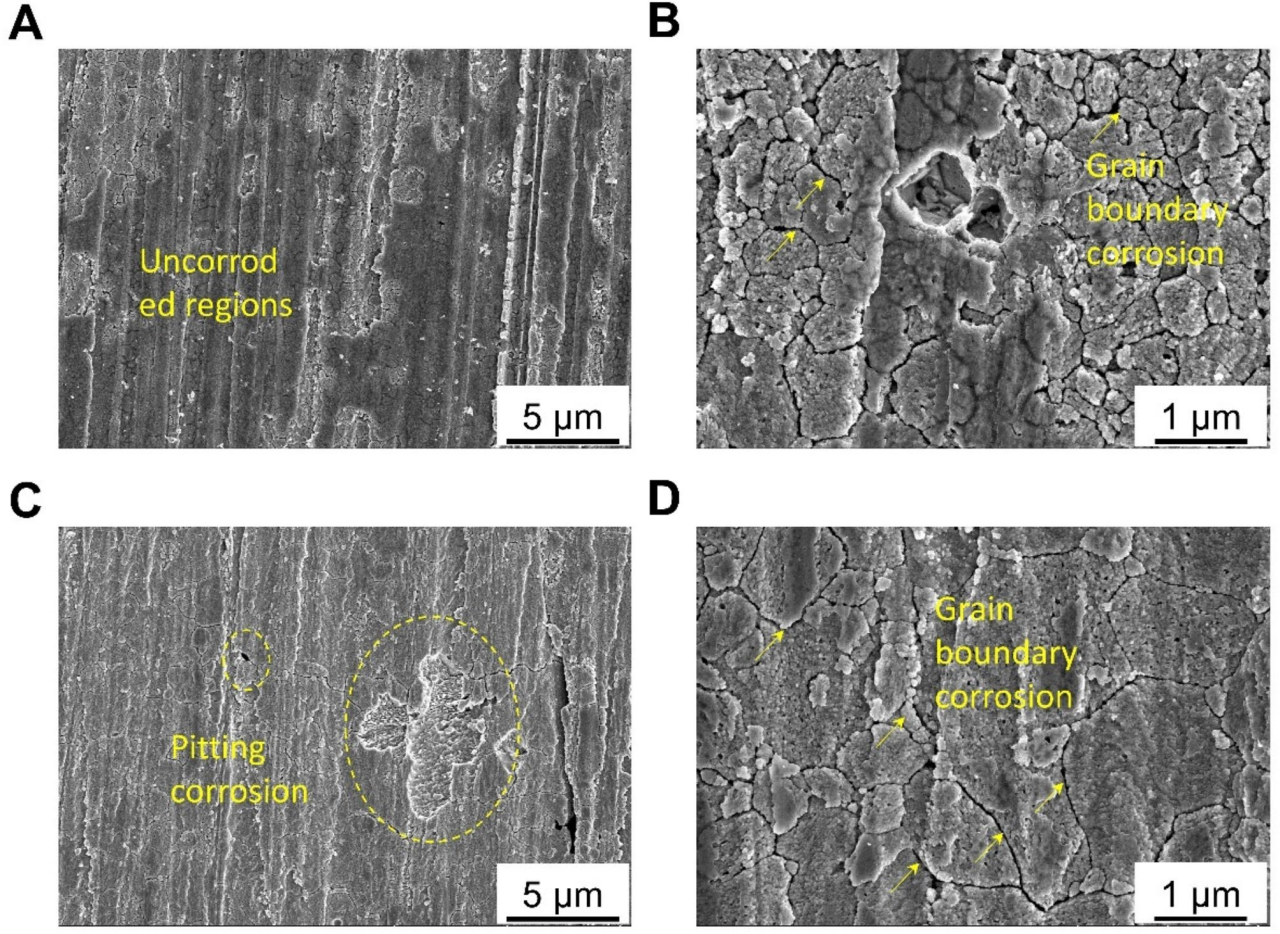
Surface morphologies of Zn-Mn alloys after removing corrosion products. (**a**) Pitting corrosion, (**b**) grain boundary corrosion in Zn-Mn alloys processed at RT. (**c**) Uncorroded regions, (**d**) grain boundary corrosion in Zn-Mn alloys after processing at 200 ℃.

Figure 5 shows the corrosion morphology of Zn-Mn alloys after the removal of corrosion products. After the removal of corrosion products from the Zn-Mn alloy processed at RT (Fig. 5a and b), grain boundary corrosion regions were observed on the surface. However, in addition to these regions, numerous dark-contrast areas were found where no corrosion cracks appeared at the grain boundaries, suggesting that the degree of corrosion in these regions was minimal. As illustrated in Fig. 5c and d, corrosion pits were present on the surface of the Zn-Mn alloy processed at 200 °C, with some pits exceeding 5 μm in size and a limited number of corrosion pits overall. High-magnification observations revealed cracks at the grain boundaries between the DRXed grains, indicating that the corrosion modes of the Zn-Mn alloys processed at 200 °C were primarily dominated by grain boundary corrosion, accompanied by pitting corrosion.

**Fig. 6:**
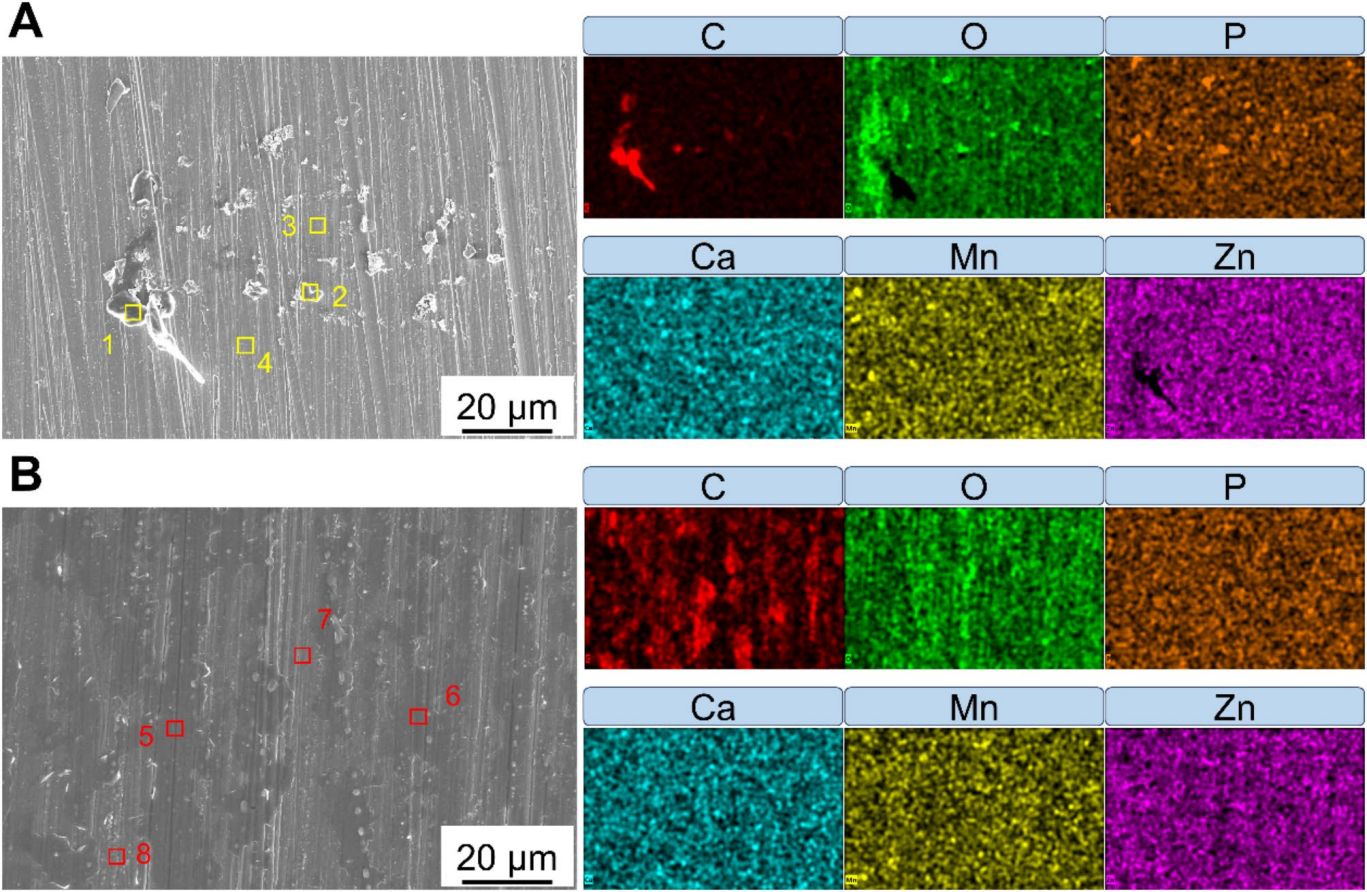
Morphologies of corrosion products. Corrosion products and the corresponding EDS mapping in Zn-Mn alloys after processing at (**a**) RT, (**b**) 200 ℃.

Figure 6a and b demonstrate that the surfaces of the Zn-Mn alloys processed at different temperatures are covered with corrosion products after 30 days of immersion. The morphology of these corrosion products was predominantly particle-like. Energy-dispersive spectroscopy (EDS) mapping and point analysis revealed a concentration of carbon (C) in these corrosion products, as shown in Table 3. In contrast, other regions exhibited significantly lower C content, often falling below the oxygen (O) content. The elevated C levels primarily resulted from the deposition of Zn_5_(CO_3_)_2_(OH)_6_ compounds according to the previous studies^20,28,29^.

Notably, the C-rich regions on the surface of the Zn-Mn alloy (200 °C) were more pronounced, with the morphology of the corrosion products shifting from a particle-like to a bulk appearance. EDS point analysis of the corrosion products indicated no significant differences in chemical composition between the two alloys. This suggested that the types of corrosion products formed for both alloys during the immersion experiments were similar. Furthermore, no obvious enrichment of manganese (Mn) was detected in the EDS results for the corrosion products, which may be attributed to the small size of the precipitated phase.

**Table 3:** Chemical compositions of corrosion products.

Areas	Weight ratio (wt%)					
	C	O	*P*	Ca	Mn	Zn
1	61.32	10.83	1.04	0.51	0.32	25.98
2	66.55	19.37	/	0.58	/	13.50
3	5.63	9.91	/	/	/	84.46
4	6.89	10.51	/	0.40	/	82.21
5	54.61	6.01	/	/	/	39.38
6	52.94	6.08	/	/	/	40.99
7	8.22	12.23	1.11	0.33	/	78.11
8	4.91	7.75	/	/	/	87.34

**Fig. 7:**
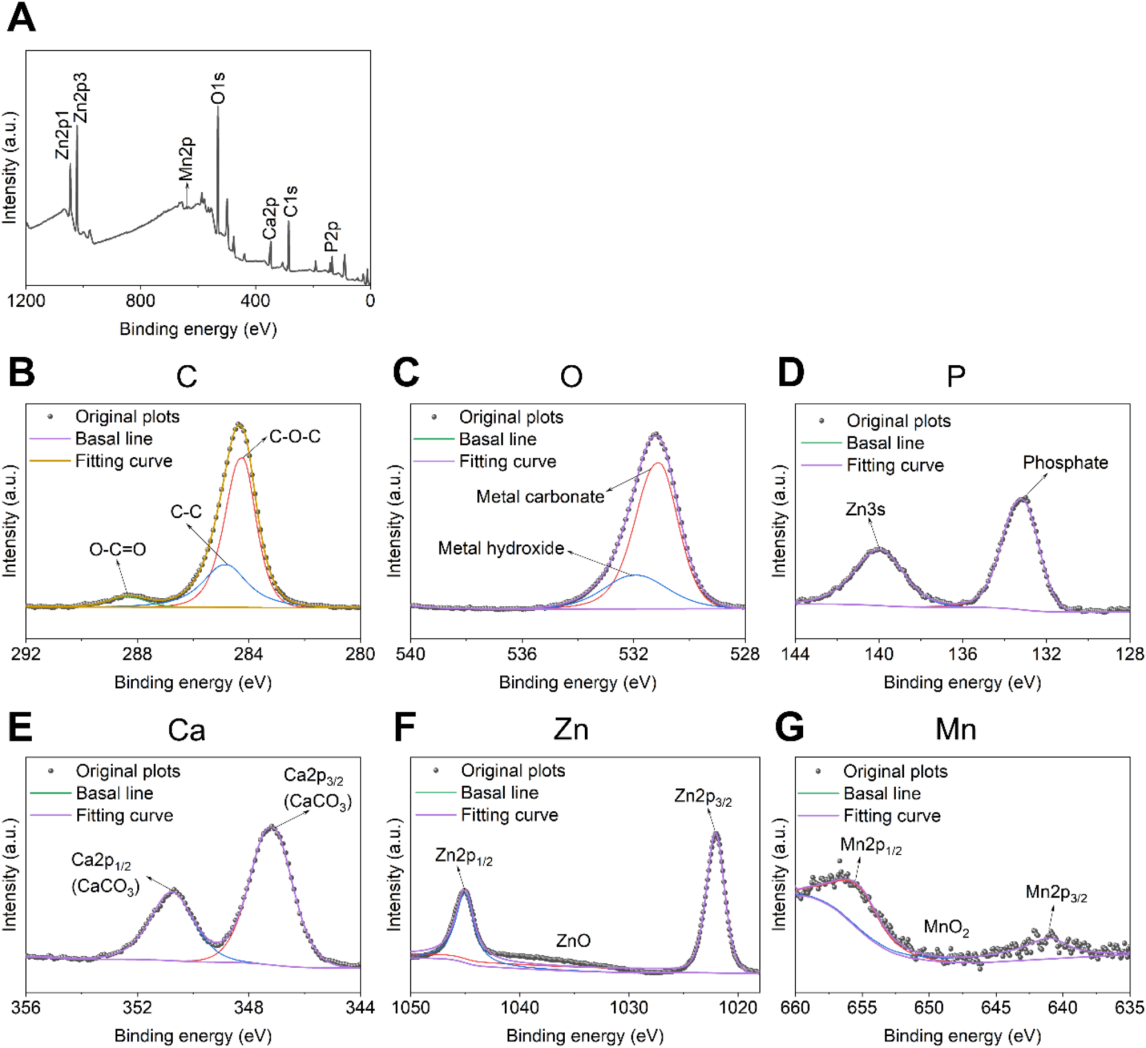
XPS analysis of corrosion products in Zn-Mn alloys processed at 200 ℃. (**a**) Survey spectrum. Narrow spectrum of (**b**) C, (**c**) O, (**d**) P, (**e**) Ca, (**f**) Zn, (**g**) Mn.

X-ray photoelectron spectroscopy (XPS) was employed to analyze the components of corrosion products in the Zn-Mn alloy processed at room temperature (RT), as shown in Fig. 7. The survey spectrum revealed that the primary chemical elements present in the corrosion products of both alloys were carbon (C), oxygen (O), phosphorus (P), calcium (Ca), manganese (Mn), and zinc (Zn). The C1s spectrum indicated the formation of carbonates during immersion, a finding further supported by the O1s and Ca2p narrow spectra. Additionally, the O1s narrow spectrum confirmed the presence of metal oxides in the corrosion products, specifically zinc oxide and manganese dioxide, which were identified in the Zn2p and Mn2p narrow spectra, respectively. The results from the P2p narrow spectrum indicated the formation of phosphates, attributed to the deposition of compounds formed by phosphate ions in the solution onto the surface of the sample.

### Biocompatibility of Zn-Mn alloys

**Fig. 8:**
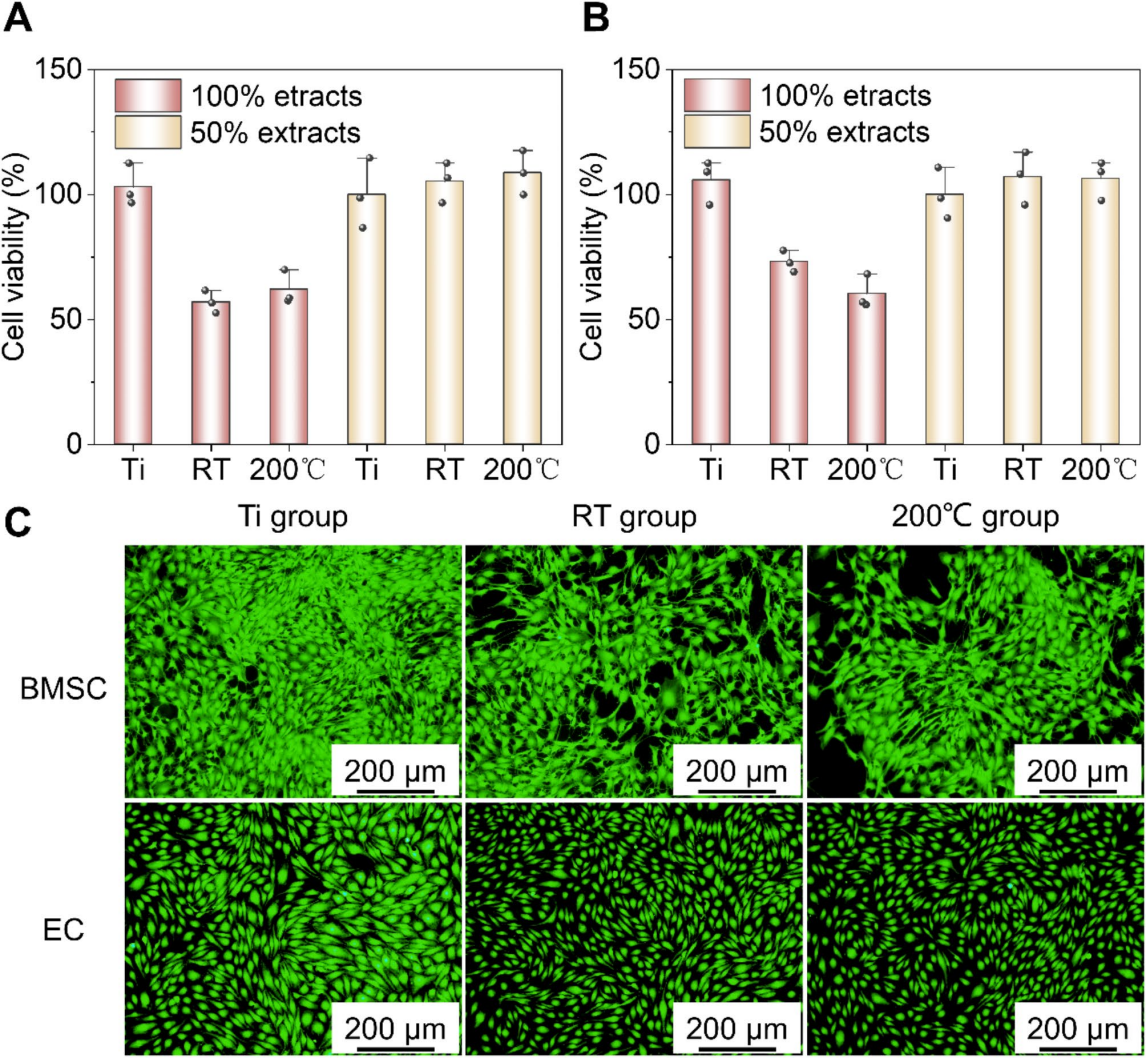
Cytocompatibility of Zn alloys. Cell viability of (**a**) BMSC and (**b**) EC. (**c**) Live/dead staining images.

Figure 8a illustrates the viability of bone mesenchymal stem cells (BMSCs) after a 72-hour incubation in 50% and 100% extracts of Zn-Mn alloys. The results indicated that higher extract concentrations correlate with lower cell viability; specifically, cell viability fell below 75% for both alloys at 100% extracts. In contrast, when the extract concentration was diluted to 50%, cell viability values increased above 90%, suggesting minimal or negligible cytotoxicity.

Figure 8b presents the viability of endothelial cells (ECs) after a similar incubation period. In the presence of 50% extracts, the cell viability remained above 100%, indicating a promotion of cell proliferation. However, at 100% extracts, cell viability for both alloys dropped below 75%. Notably, for the Zn-Mn alloy processed at room temperature (RT), the EC viability was higher than that of BMSCs at 100% extracts. Conversely, for the Zn-Mn alloy processed at 200 °C, EC viability was 56.7%, which closely matched the BMSC viability of 57.0% in 100% extracts.

Figure 8c depicts live/dead staining results for BMSCs and ECs cultured in 50% extracts for 72 h, where live cells are indicated in green. In this case, the living cell densities for both Zn alloys were comparable, though slightly lower than that of the titanium (Ti) group. Despite having lower living cell densities than the Ti group, the Zn alloy groups displayed favorable growth morphology among the cells.

**Fig. 9:**
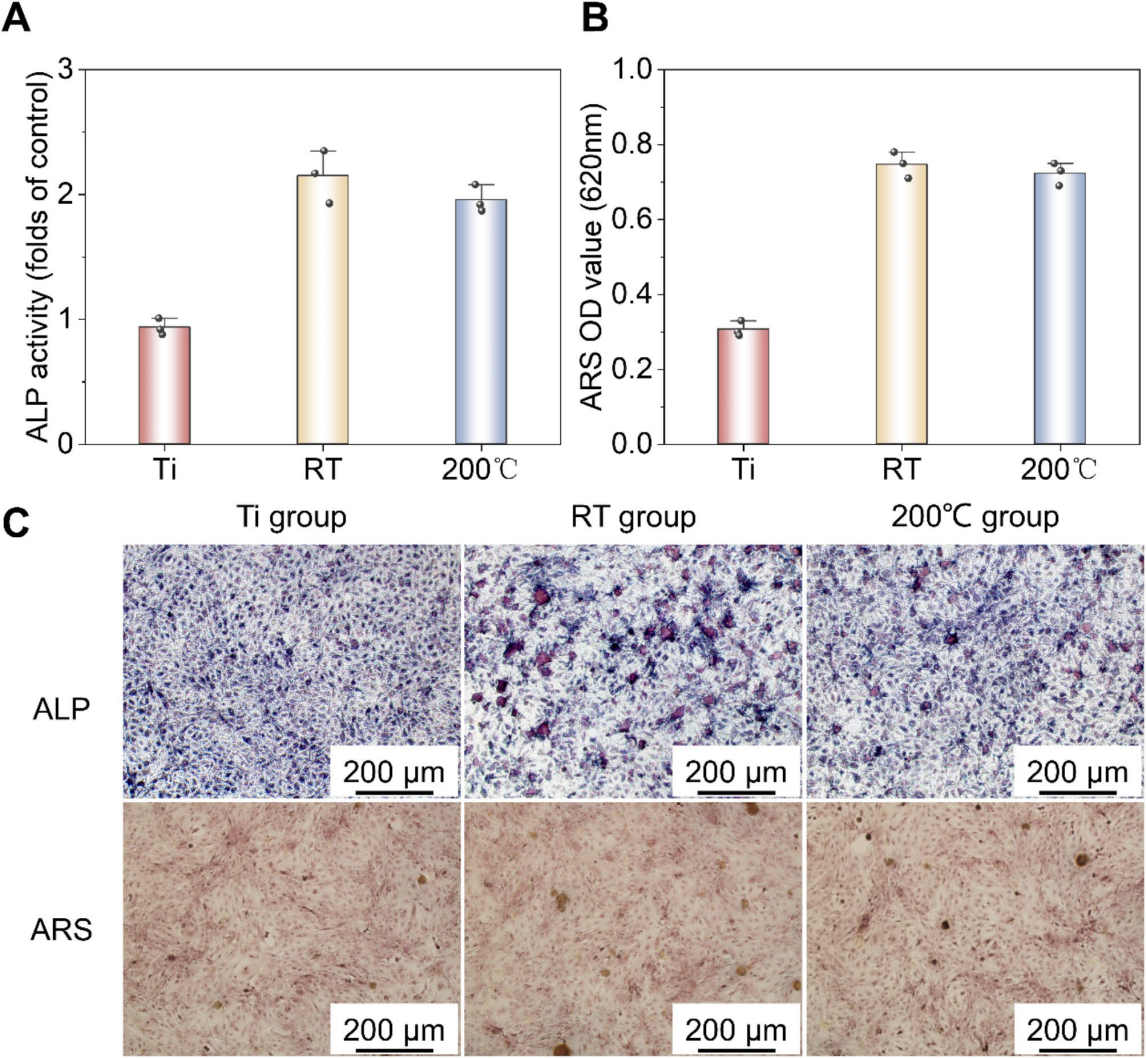
Osteogenic properties of the studied Zn alloys. (**a**) ALP activity. (**b**) ARS analysis of BMSC cells. (**c**) ALP and ARS staining images.

The results from the cell viability experiments indicated that BMSCs cultured in 50% extracts of both Zn alloys exhibited favorable morphologies and living cell densities. Consequently, these extracts were employed to further assess their effects on the osteogenic differentiation ability of BMSCs. Alkaline phosphatase (ALP) served as an early marker for osteoblast differentiation. Figure 9a displays ALP and alizarin red staining images of BMSCs incubated in 50% extracts for 7 and 14 days. In the ALP staining results, deeper purple coloration signified stronger ALP expression. After 7 days of cultivation, the ALP expression levels for the Zn-Mn alloys (both RT and 200 °C) were higher than that of the Ti control group, with the Zn-Mn alloy processed at room temperature (RT) showing the highest expression. Quantitative analysis of ALP activity is presented in Fig. 9b. After 7 days of cultivation, ALP activities were ranked as follows: Zn-Mn alloy (RT) > Zn-Mn alloy (200 °C) > Ti group. These findings indicate that the 50% extracts of both Zn alloys significantly promoted the osteogenic differentiation of BMSCs.

Mineralized nodules served as markers of osteoblast differentiation and maturation. In Fig. 9a, the staining results show mineralized nodules in BMSCs cultured in extracts over 14 days, with the nodules staining orange-red. When compared to the Ti control group, cells in the 50% extracts of both Zn-Mn alloys displayed a deeper orange-red hue, indicating a higher presence of mineralized nodules in osteoblasts. As shown in Fig. 9c, the number of mineralized nodules in the 25% extracts followed this order: Zn-Mn alloy (RT) ~ Zn-Mn alloy (200 °C) > Ti group. This suggested that the 25% extract concentration can significantly enhance cell matrix mineralization and effectively support osteogenic cell differentiation.

## Discussion

### Precipitates induce high elongation

The tensile curve results indicated that the elongation of the Zn-Mn alloy processed at room temperature (RT) was greater than that of the Zn-Mn alloy processed at 200 °C. This increase in elongation can be primarily attributed to grain refinement and the formation of nanoscale precipitates. Specifically, grain refinement enhanced grain boundary density, which was measured via EBSD at 1.83 × 10^6^ /m for the Zn-Mn alloy (RT) compared to 0.94 × 10^6^ /m for the alloy processed at 200 °C. Given the low melting point of Zn (692 K), the tensile test temperature of 295 K is 0.43 times the melting point, situating it within the intermediate temperature range of 25–27 °C^30–32^. At this temperature, the increased grain boundary density facilitates grain boundary slip through grain boundary diffusion, resulting in enhanced elongation^33^. The tensile curves for both alloys exhibited significant softening during the plastic flow stage, suggesting minimal dislocation proliferation within the grains during tension at RT. This decrease in dislocation density can be attributed to two factors: first, the small grain size hinders dislocation activation, a characteristic common in nanocrystalline materials^34,35^. Second, dynamic recovery leads to the annihilation and rearrangement of dislocations into low-angle grain boundaries^36^. The experimentally measured low-angle grain boundary density for the Zn-Mn alloy (RT) was 0.28 × 10^6^ /m, which was higher than the 0.24 × 10^6^ /m measured for the Zn-Mn alloy (200 °C), indicating that reduced grain size promotes dislocation annihilation.

Furthermore, a significant presence of nanoscale precipitates (MnZn_13_ phase) was observed in the microstructure of the Zn-Mn alloy (RT). This second phase interacted with dislocations during tension, operating via the Orowan mechanism (for undeformed particles) and the shearing mechanism (for deformed particles)^37^. The MnZn_13_ phase in this study comprised deformed particles, leading to dislocation accumulation at the interface and subsequent plastic deformation of the precipitates. TEM results indicated that the deformation of the MnZn_13_ phase was predominantly governed by twinning. The plastic deformation of these precipitates served two critical functions: it alleviated accumulated strain, thus mitigating the deformation incompatibility associated with the modulus difference between the Zn matrix and the MnZn_13_ phase, and it effectively resolved stress concentrations at the matrix-precipitate interface, reducing the likelihood of crack initiation. In conclusion, the high elongation observed in the Zn-Mn alloys processed at RT can be primarily ascribed to grain refinement, which enhanced grain boundary slip and facilitated dislocation annihilation through dynamic recovery. Additionally, the plastic deformation of the precipitates alleviated stress concentrations at the interface, contributing to the overall mechanical performance.

### Precipitation strengthening

Research on ultrafine-grained Zn-Mn alloys has demonstrated that grain refinement can lead to a reduction in strength^24^. This phenomenon is primarily attributed to insufficient dislocation accommodation within the grains and the observation that the strength of grain boundaries is generally lower than that of the grains themselves. In this study, the grain size of the Zn-Mn alloy processed at room temperature (RT) was smaller than that of the alloy processed at 200 °C; however, the RT alloy exhibited higher tensile strength. This enhancement in strength was mainly due to the abundant nanoscale precipitates present in the microstructure. Previous studies have confirmed that the MnZn_13_ phase exhibits greater hardness compared to the Zn matrix, as shown through nanoindentation experiments^23^. The microstructure of the Zn-Mn alloy (RT) consisted of two phases with distinct mechanical properties, whereas the microstructure of the Zn-Mn alloy (200 °C) was composed entirely of dynamically recrystallized (DRXed) grains. Unlike the homogeneous fine-grained microstructure, the heterogeneous structure of the Zn-Mn alloy (RT), which included a second phase, generated back-stress strengthening during tension^38,39^. This back-stress originated from the differing deformation behaviors of the Zn matrix and the MnZn_13_ phase. Typically, the softer Zn matrix underwent preferential plastic deformation under applied stress, leading to dislocation accumulation around the harder MnZn_13_ phase. The pile-up of dislocations in the vicinity of the MnZn_13_ phase generated long-range stress, which interacted with newly generated dislocations, thereby enhancing strength. Consequently, the significant presence of nanoscale precipitates in the Zn-Mn alloys (RT) contributed to strength enhancement while maintaining continuous grain refinement.

### Influence of precipitates on corrosion

The results of electrochemical and immersion experiments indicated that the corrosion rate of the Zn-Mn alloy processed at room temperature (RT) was slightly lower than that of the Zn-Mn alloy processed at 200 °C. Surface morphology analysis following the removal of corrosion products revealed that the corrosion mode of the Zn-Mn alloy (RT) was primarily characterized by grain boundary corrosion, while the corrosion mode of the Zn-Mn alloy (200 °C) was dominated by grain boundary corrosion accompanied by pitting corrosion. This difference in corrosion behavior can be attributed to variations in microstructure. In the Zn-Mn alloys processed at 200 °C, the homogeneous ultrafine-grained microstructure resulted in minimal potential differences within the material. The disordered structure of the grain boundaries, along with the enrichment of solute elements, contributed to the formation of localized potential differences between the grain boundaries and the grain interior. This phenomenon led to preferential corrosion of the grain boundaries, which in turn generated grain boundary corrosion cracks. As immersion time increases, these localized corrosion cracks expanded, subsequently leading to larger-scale pitting corrosion.

In contrast to the Zn-Mn alloys processed at 200 °C, the Zn-Mn alloys processed at room temperature (RT) contained a significant number of MnZn_13_ phases within their microstructures. This resulted in a potential difference between these second phases and the substrate, leading to the formation of a localized microcell structure during the corrosion process, which promoted galvanic coupling corrosion. Although the standard electrode potential of manganese is lower than that of zinc, the potential of the MnZn_13_ phase is higher than that of Zn^29,40^. This indicated that the MnZn_13_ phase acted as the cathodic component in the corrosion process. Additionally, in magnesium alloys, incorporating a high-potential second phase has been shown to effectively enhance corrosion resistance^41,42^. Consequently, the lower corrosion rate observed in the Zn-Mn alloy (RT) can be attributed to the presence of the high-potential MnZn_13_ phase.

## Conclusion

In our study, the formation of nanoscale MnZn_13_ phases was controlled by varying the equal channel angular pressing (ECAP) temperature. Microstructural analysis revealed that the Zn-Mn alloy containing the MnZn_13_ phase exhibits smaller dynamically recrystallized (DRXed) grains compared to the Zn-Mn alloy without the MnZn_13_ phase. This refined microstructure enhanced both mechanical properties and corrosion resistance. The improvement in elongation was attributed to grain boundary sliding and the presence of deformation twins in the MnZn_13_ phase. Additionally, grain boundary corrosion in the Zn-Mn alloy with the MnZn_13_ phase was less pronounced. In contrast, the corrosion mode of the Zn-Mn alloys lacking the MnZn_13_ phase was primarily characterized by grain boundary corrosion, accompanied by pitting corrosion. The enhanced corrosion resistance observed in the presence of the MnZn_13_ phase arose from its higher potential relative to the zinc matrix. Notably, Zn-Mn alloys with and without the MnZn_13_ phase demonstrated comparable cytocompatibility and osteogenic properties. Our findings offered an effective approach to regulate the mechanical properties and corrosion resistance of Zn alloys by controlling precipitation.

## Data Availability

The datasets used and analysed during the current study available from the corresponding author on reasonable request.
